# Effects of different therapy regimens to increase final adult height in males at advanced bone age with idiopathic short stature

**DOI:** 10.1186/s12887-023-04429-9

**Published:** 2023-12-05

**Authors:** Wei Wang, Yan Wang, Ya Xiao, Niuniu Cao, Yifan Wang

**Affiliations:** 1 Pediatric Endocrinology Clinic, Zhengzhou University Third Hospital and Henan Province Women and Children’s Hospital, Zhengzhou, 450000 China; 2 Zhengzhou University Third Hospital and Henan Province Women and Children’s Hospital, Zhengzhou, 450000 China

**Keywords:** Idiopathic short stature, Advanced bone age, Recombinant human growth hormone, Gonadotropin-releasing hormone analog, Aromatase inhibitors

## Abstract

**Background:**

This retrospective study explored the effect on adult height of a combination of recombinant human growth hormone (rhGH) and aromatase inhibitors (AIs), or rhGH and a gonadotropin-releasing hormone analog (GnRHa), and compared their effects with rhGH alone in males at advanced bone age with idiopathic short stature (ISS).

**Methods:**

In this retrospective study, rhGH or rhGH combined with GnRHa or rhGH combined with AI therapy was given to males with advanced bone age (13–15 years) and diagnosed with ISS. The patients were followed to assess their adult height.

**Results:**

(1) A total of 68 patients were reviewed; 22 males were treated with rhGH for 24.9 ± 4.47 months, 22 males were treated with GnRHa + rhGH for 34.1 ± 3.36 months, and 24 males were treated with AI + RHGH for 22.7 ± 2.49 months. (2) Before treatment, the HtSDS-CA for the three groups were -1.04 ± 0.95, -1.23 ± 1.06, and -0.85 ± 0.98, respectively, and the HtSDS-BA were -2.14 ± 0.29, -2.14 ± 0.21, and-2.26 ± 0.31, respectively. The target heights for each group were 169.7 ± 4.0 cm, 169.7 ± 3.9 cm, and 169.1 ± 3.9 cm, respectively. The predicted adult heights were 161.7 ± 3.35 cm, 162.3 ± 1.75 cm, and 161.6 ± 2.89 cm, respectively. (3) After treatment, the HtSDS-CA for the rhGH group increased by 1.30 ± 0.58, and the HtSDS-BA increased by 2.00 ± 0.27. For the GnRHa + rhGH group, the HtSDS-CA and HtSDS-BA increased by1.42 ± 0.73and 2.74 ± 0.28, respectively. The AI + RHGH group increased by1.39 ± 0.64 and 2.76 ± 0.31, respectively. (4) There was no significant difference between the adult height (170.9 ± 0.7 cm) and target height for the rhGH group (*P* > 0.05), but the adult heights for the GnRHa + rhGH and AI + RHGH groups (173.2 ± 1.5 cm and 173.5 ± 1.0 cm, respectively, *P* > 0.05) were higher than the target height (*P* < 0.05). (5) Compared with the predicted adult height, the adult heights for the three groups improved significantly (*P* < 0.05). (6) No severe adverse reactions during the treatment occurred in any of the children. However, the total incidence of side effects in the three groups was significant (χ^2^ = 20.433, *P* = 0.00).

**Conclusion:**

Different therapeutic approaches have been investigated to improve the final adult height of males at advanced bone ages with ISS, and the optimal strategy remains controversial. In children at advanced bone ages with ISS, clinicians should carefully consider the advantages and disadvantages prior to treatment.

## Introduction

Recombinant human growth hormone (rhGH) therapy is the most effective treatment for idiopathic short stature (ISS) in children [1]. When given to prepubertal children, rhGH can successfully increase the growth velocity and adult height. However, due to early puberty onset and (or) delayed initial consultation, some adolescent children with ISS experience accelerated linear bone growth at the time of treatment [2]. It is generally agreed that an advanced stage of bone age in pubescent boys limits the application window in which to treat with rhGH [3, 4]. Thus, to maximize the adult height (AHt), endocrinologists have sought therapeutic alternatives to delay bone maturation and epiphyseal fusion.

Estrogen is a well-known regulator of endochondral bone growth and plays an essential role in controlling longitudinal bone growth during puberty by promoting the closure of the epiphyses [5, 6]. GnRHa, which promotes the regression of sexual characteristics by competing for receptors and inhibiting gonadotropin secretion, can effectively delay bone maturation and improve the adult height achieved by children [7, 8]. However, blocking estrogen production might delay epiphyseal fusion, potentially leading to a prolonged period of longitudinal growth and an increased final AHt. Aromatase inhibitors (AIs) block the activity of the aromatase enzyme CYP 19, which promotes the production of estrogen from androgens [9, 10]. Several studies have suggested that monotherapy with GnRHa or AIs can have a small and variable effect on adult height for short stature at advanced bone age and is generally not recommended [11, 12]. However, combination therapy with rhGH and GnRHa or rhGH and AIs has potential value [5, 13, 14]. Additionally, whether the AHt can reach the normal range after therapy is considered the gold standard for evaluating therapy efficacy. However, to date, few clinical studies have been published. The primary purpose of this study was to explore the effect of different treatments on increasing the AHt of boys at advanced bone age with ISS.

## Methods

### Participants

All ISS adolescent males attending the Pediatric Endocrinology Clinic of the Third Affiliated Hospital of Zhengzhou University and being treated with rhGH or GnRHa + rhGH or AI + rhGH therapy were considered candidates for this review. The participants were required to meet the following inclusion criteria: ① aged 11.0 to 16.0 years; ② bone age above or equal to 13 years but below or equal to 15 years; ③ a testis volume greater than 4 ml; ④ duration of treatment was longer than one year, therapy had been discontinued, and the patient had achieved their final AHt.

ISS was defined based on the normal height of children in nine Chinese cities in 2005. The height should have been at least 2 standard deviations or the third percentile below the mean for the given age. In addition, the peak GH level should be > 10 ng/mL with a GH stimulation test. Although some adolescents did not meet the definition of ISS at the time of entry into the study (because their height was not <  − 2SD), their predicted height calculated based on the current bone age was so poor as to indicate that their height would be below—2 SD when they reach adulthood.

Exclusion criteria included chronic organic diseases, growth hormone deficiency (GHD), small for gestational age at birth, chromosomal abnormalities, skeletal dysplasia, genetic metabolic diseases, psychosocial deprivation, and the use of medication that interfered with GH secretion or action. Adult height (AHt) was confirmed when the growth velocity was < 1.5 cm/year or the bone age was determined to be 17 years. This study was approved by the Institutional Review Board at the Third Affiliated Hospital of Zhengzhou University (approval number 2022–128-01).

### Study design

A GH stimulation test was performed to exclude GH deficiency using 0.15 mg/m^2^ of oral L-dopa and 50 mcg/kg of insulin IV, not exceeding 1 mg. The rhGH was administered subcutaneously at a dose of 0.05 mg/kg/day for six out of seven days each week. (i) rhGH was given alone, or (ii) with GnRHa (leuprorelin) administered monthly as an intramuscular dose of 3.75 mg, or (iii) with an AI (letrozole) administered orally at a dose of 2.5 mg/day.

The data collected included height, weight, secondary sexual characteristics, testicular volume (measured using a testicle mold), liver, kidney, and heart function, blood glucose, thyroid function, and IGF-1. The assessments were recorded at three- to six-month intervals. Experienced endocrinologists evaluated a left hand and wrist radiograph based on the Greulich and Pyle atlas at six-month intervals.

### Reference index

The HtSDS was calculated from the table of standard deviations of height in children aged 0 to 18 years in China [15, 16]. The standard deviation score for the chronological age and height (HtSDS-CA) = (patient height − the average height for same age and sex)/standard deviation of the height for the same age and sex. The standard deviation score for the bone age and height (HtSDS-BA) = (patient height − the average height for the same bone age and sex)/standard deviation of the height for same bone age and sex. Finally, the target height (THt, cm) = (father’s height + mother’s height + 13)/2. The predicted adult height (PAH) was calculated using the Bayley-Pinneau method [17].

### Data analysis

Data were expressed as means ± SD, and the statistical analyses were conducted using SPSS 26.0 software. Differences between the three groups were assessed using one-way analysis of variance for independent samples for data that conformed to a normal distribution. A non-parametric test was used for data that exhibited a skew distribution. The rank-sum test was used to compare the same group before and after treatment, and the incidence of adverse reactions among the three groups was assessed using the chi-square test. The level of statistical significance was set at *P* < 0.05.

## Results

### Clinical characteristics of patients at the onset of treatment

Sixty-eight children were recruited for this study, and Table 1 shows the clinical characteristics of the patients at the initiation of treatment in the three groups. The average age of the boys in the three groups when the treatment started (baseline ages), their baseline height, the baseline HtSDS-CA, baseline HtSDS-BA, PAH, and THt did not exhibit any significant differences (*P* > 0.05). There also were no significant differences observed for the levels of IGF-1, TSH, and T4 (see Table 1).

**Table 1:** Clinical characteristics of patients at initiation of treatment

Clinical characteristics	rhGH *n* = 22	GnRHa + GH *n* = 22	AI + GH *n* = 24	***P***
Baseline age (years)	**13.0 ± 0.95**	**13.3 ± 0.94**	**12.9 ± 1.03**	** > 0.05**
Baseline bone age (years)	**14.0 ± 0.58**	**14.1 ± 0.41**	**14.1 ± 0.54**	** > 0.05**
Baseline height (cm)	**151.5 ± 2.99**	**151.3 ± 2.54**	**151.1 ± 2.12**	** > 0.05**
Baseline HtSDS-CA	**-1.04 ± 0.95**	**-1.23 ± 1.06**	**-0.85 ± 0.98**	** > 0.05**
Baseline HtSDS-BA	**-2.14 ± 0.29**	**-2.14 ± 0.21**	**-2.26 ± 0.31**	** > 0.05**
PHt	**161.7 ± 3.35**	**162.3 ± 1.75**	**161.6 ± 2.89**	** > 0.05**
THt	**169.7 ± 4.0**	**169.7 ± 3.9**	**169.1 ± 3.9**	** > 0.05**
IGF-1(nmol/L)	**56.2 ± 26.9**	**54.3 ± 20.6**	**57.4 ± 39.7**	** > 0.05**
TSH (mIu/L)	**2.79 ± 1.45**	**2.21 ± 1.02**	**2.01 ± 1.20**	** > 0.05**
T4(Pmol/L)	**14.7 ± 1.7**	**14.1 ± 2.3**	**14.4 ± 1.7**	** > 0.05**

*HtSDS-CA* the standard deviation score of chronological age and height, *HtSDS-BA* the standard deviation score of bone age and height, *PHt* Predicted adult height, *THt* target Height

### Clinical characteristics of patients after treatment

**Fig. 1:**
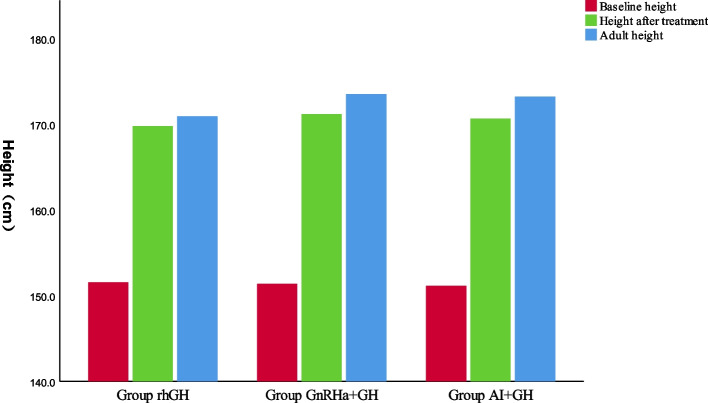
Before and after treatment, the difference of height in three groups

The duration of treatment and age for the patients in the GnRHa + rhGH group was significantly higher than the other two groups (*P* < 0.05). The bone age and HtSDS-BA after treatment were significantly different for the AI + rhGH and GnRHa + rhGH groups compared to the rhGH group (*P* < 0.05). Growth had ceased at the final follow-up visit, and the AHt for the GnRHa + rhGH and AI + rhGH groups did not exhibit any significant differences (*P* ˃ 0.05). However, both were higher than the AHt observed for the rhGH group (*P* < 0.05) (see Table 2 and Fig. 1).

**Table 2:** Clinical characteristics of patients after treatment

Clinical characteristics	rhGH *n* = 22	GnRHa + GH *n* = 22	AI + GH *n* = 24	***P***
Age^*^ (years)	**15.1 ± 1.12**^**a**^	**16.1 ± 0.94**^**ac**^	**14.8 ± 1.10**^**c**^	** < 0.05**
Bone age^*^(years)	**15.2 ± 0.53**^**ab**^	**14.1 ± 0.47**^**a**^	**14.3 ± 0.45**^**b**^	** < 0.05**
HtSDS-CA^*^	**0.12 ± 0.65**	**0.03 ± 0.34**^**c**^	**0.39 ± 0.47**^**c**^	** < 0.05**
HtSDS-BA^*^	**-0.14 ± 0.18**^**ab**^	**0.60 ± 0.29**^**a**^	**0.51 ± 0.24**^**b**^	** < 0.05**
Duration (months)	**24.9 ± 4.47**^**a**^	**34.1 ± 3.36**^**ac**^	**22.7 ± 2.49**^**c**^	** < 0.05**
Height^*^(cm)	**169.8 ± 0.83**	**171.2 ± 0.81**	**170.6 ± 0.75**	** > 0.05**
AHt	**170.9 ± 0.7**^**ab**^	**173.5 ± 1.0**^**a**^	**173.2 ± 1.5**^**b**^	** < 0.05**
AHtSDS	**-0.29 ± 0.1**^**ab**^	**0.07 ± 0.2**^**a**^	**0.09 ± 0.2**^**b**^	** < 0.05**
IGF-1^*^ (nmol/L)	**62.1 ± 19.6**^**b**^	**74.6 ± 23.4**	**80.9 ± 20.8**^**b**^	** < 0.05**
TSH^*^ (mIu/L)	**1.92 ± 0.96**	**1.65 ± 0.57**	**1.69 ± 0.77**	** > 0.05**
T4^*^ (Pmol/L)	**15.9 ± 2.2**	**14.5 ± 3.1**	**15.7 ± 3.2**	** > 0.05**

^*^: when therapy discontinued. HtSDS-CA: the standard deviation score of chronological age and height, HtSDS-BA:the standard deviation score of bone age and height, AHt: Adult Height, AHtSDS: AHt standard deviation score

^a^*P* < 0.05(Comparison between GH group and GnRHa + GH group),^b^*P* < 0.05(Comparison between GH group and AI + GH group), ^c^*P* < 0.05(Comparison between GnRHa + GH group and AI + GH group)

### The disparity in the standard deviation scores for height and the height differences in the three groups

**Fig. 2:**
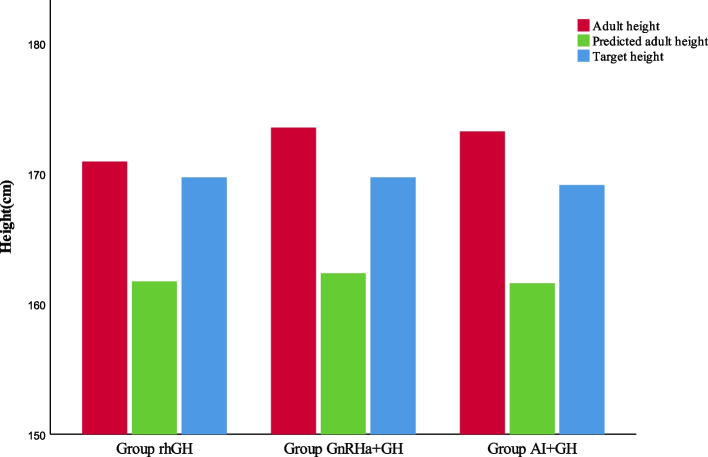
Comparison adult height and target height and predicted adult height after different treatments

**Fig. 3:**
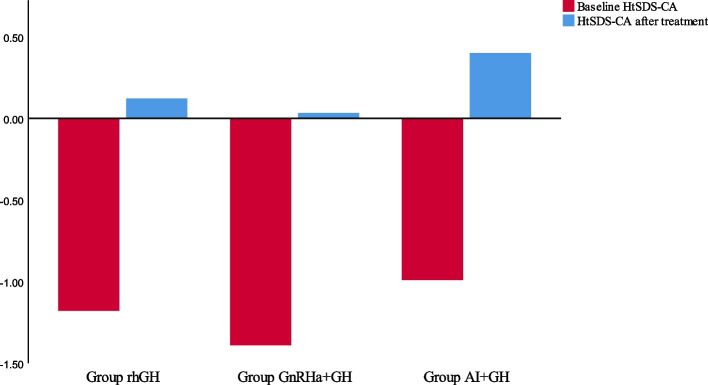
Change in the standard deviation score of chronological age and height

**Fig. 4:**
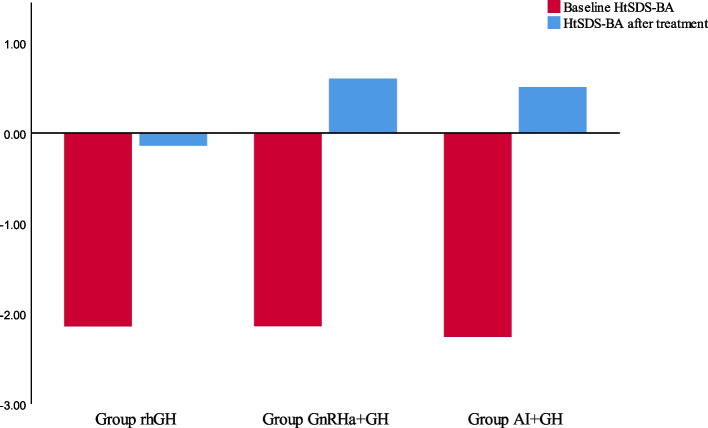
Change in the standard deviation score of bone age and height

After treatment, the HtSDS-CA for the rhGH group was 0.12 ± 0.65, revealing an increase of 1.30 ± 0.58 (*P* < 0.05) compared with the baseline HtSDS-CA. The HtSDS-BA exceeded the baseline HtSDS-BA by2.00 ± 0.27 (*P* < 0.05). The AHt was 170.9 ± 0.7 cm, and the disparity was 1.21 ± 3.78 cm compared with the THt (*P* > 0.05), but significantly exceeded PAH by approximately 9.02 cm (*P* < 0.05) (see Table 3 and Figs. 2, 3, and 4).

**Table 3:** Before and after treatment, the standard deviation score of height and the difference of height in three groups

	rhGH	***P***_1_	GnRHa + GH	***P***_2_	AI + GH	***P***_3_
HtSDS-CA^a^-HtSDS-CA	1.30 ± 0.58	< 0.05	1.42 ± 0.73	< 0.05	1.39 ± 0.64	< 0.05
HtSDS-BA^a^-HtSDS-BA	2.00 ± 0.27	< 0.05	2.74 ± 0.28	< 0.05	2.76 ± 0.31	< 0.05
AHtSDS-THtSDS	0.20 ± 0.63	> 0.05	0.63 ± 0.71	< 0.05	0.69 ± 0.69	< 0.05
AHtSDS-PHtSDS	1.53 ± 0.53	< 0.05	1.86 ± 0.29	< 0.05	1.94 ± 0.38	< 0.05
AHt-THt (cm)	1.21 ± 3.78	> 0.05	3.80 ± 4.28	< 0.05	4.13 ± 4.12	< 0.05
AHt-PHt (cm)	9.20 ± 3.19	< 0.05	11.16 ± 1.72	< 0.05	11.67 ± 2.32	< 0.05
PHt-THt (cm)	-7.99 ± 4.92	< 0.05	-7.36 ± 4.39	< 0.05	-7.54 ± 4.73	< 0.05

*HtSDS-CA* the standard deviation score of chronological age and height, *HtSDS-BA* the standard deviation score of bone age and height, *AHt* Adult height, *AHtSDS* adult height standard deviation score, *PHt* Predicted adult height, *THt* target Height, *PHtSDS* Predicted adult height standard deviation score, *THt* target height standard deviation score

^a^: when therapy discontinued

The HtSDS-CA in the GnRHa + rhGH group was 0.03 ± 0.34 and increased to 1.42 ± 0.73 (*P* < 0.05), compared with the baseline HtSDS-CA. The HtSDS-BA exceeded the baseline HtSDS-BA by 2.74 ± 0.28 (*P* < 0.05). The AHt was 173.5 ± 1.0 cm, which had increased by 3.80 ± 4.28 cm compared with the THt and significantly exceeded PAH by 11.16 ± 1.72 cm (*P* < 0.05) (see Table 3 and Figs. 2, 3, and 4).

The HtSDS-CA in the AI + RHGH group significantly increased by 1.39 ± 0.64 after treatment (*P* < 0.05), and the HtSDS-BA exceeded the baseline HtSDS-BA by 2.76 ± 0.31. The AHt was 173.2 ± 1.5 cm, which showed a significant increase of 4.13 ± 4.12 cm compared with the THt, and 11.67 ± 2.32 cm compared with the PAH (see Table 3 and Figs. 2, 3 and 4).

### Growth velocity changes in the three groups within 18 months

**Fig. 5:**
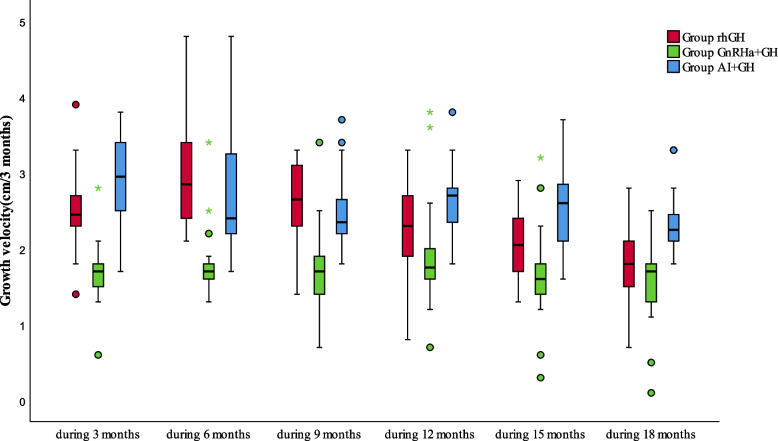
Comparison of growth velocity in three groups

Data derived from the shortest treatment time for the three groups were used to compare the growth velocities at 18 months, as seen in Table 4. The growth velocity (GV) for the rhGH group was higher in the first year and similar to that of the AI + rhGH group, but higher than the GnRHa + rhGH group (*P* < 0.05), and then gradually declined. The GV decreased significantly in the third half of the year, with no differences compared to the GnRHa + rhGH group (*P* > 0.05). The GV for the AI + rhGH group was maintained at a relatively high level during the treatment course (details provided in Fig. 5).

**Table 4:** Growth velocity during 18 months

Group	GV (cm/3 months)		
	The first half year	The second half year	The third half year
Group rhGH	**2.67 ± 0.44**^**a**^	**2.56 ± 0.38**^**a**^	**1.94 ± 0.37**^**b**^
Group GnRHa + GH	**1.90 ± 0.34**^**ac**^	**1.84 ± 0.39**^**ac**^	**1.64 ± 0.42**^**c**^
Group AI + GH	**2.79 ± 0.43**^**c**^	**2.62 ± 0.34**^**c**^	**2.53 ± 0.38**^**bc**^
***P***	** < 0.05**	** < 0.05**	** < 0.05**

*GV* growth velocity

^a^*P* < 0.05(Comparison between GH group and GnRHa + GH group),^b^*P* < 0.05(Comparison between GH group and AI + GH group), ^c^*P* < 0.05(Comparison between GnRHa + GH group and AI + GH group)

### Adverse drug reactions during treatment

Eleven cases (48.5%),in AI + rhGH group, showed hyperandrogenic symptoms, including acne, increased sebum secretion or oily skin, and beard hair growth. However, after the termination of the letrozole treatment, the hyperandrogenic effects disappeared, the blood testosterone levels returned to normal, and the testicular volume in adulthood was normal. A transient increase in insulin occurred in two patients but returned to normal after stopping treatment for two weeks. One patient in the rhGH group developed hypothyroidism but returned to normal with thyroxine supplementation. No severe adverse reactions were observed in any of the children, but the total incidence of all side effects in the three groups was significantly different (χ^2^ = 20.433, *P* = 0.00). There were no significant changes in liver function, thyroid hormone levels, fasting insulin, or blood glucose during the treatment.

## Discussion

Adult height is considered the gold standard for evaluating the efficacy of therapies used to treat short stature in children. Our study showed that with rhGH treatment alone, HtSDS-CA and HtSDS-BA increased by 1.30 ± 0.58 and 2.00 ± 0.27, respectively. Whereas GnRHa + rhGH treatment increased HtSDS-CA and HtSDS-BA by1.42 ± 0.73 and 2.74 ± 0.28, respectively, and AI + rhGH treatment increased HtSDS-CA and HtSDS-BA by 1.39 ± 0.64 and 2.76 ± 0.31, respectively. Compared to the predicted adult height, the adult heights improved for all patients. However, compared with the THt, patients treated with rhGH alone did not exceed the AHt (the disparity was 1.21 ± 3.78 cm). The GnRHa + rhGH and AI + rhGH treated patients exceeded this by 3.80 cm and 4.13 cm, respectively. These results demonstrated that all therapy regimens could significantly improve AHt in male children at advanced bone age with idiopathic short stature.

Our study indicated that the growth velocity exhibited by patients treated with rhGH alone gradually decreased with the development of puberty, and while the AHt of the rhGH group did exceed the predicted adult, it could not attain the target height. The underlying mechanism might be that elevations in sex hormones gradually accelerated epiphyseal maturation and closure during puberty, resulting in an increased speed of epiphyseal fusion. Therefore, some studies suggest that the dose of rhGH should be increased when puberty begins, which has been shown to be beneficial [18]. However, some scholars have found that higher doses of rhGH can advance bone age and puberty [19, 20]. In summary, delaying epiphyseal progression to increase linear growth time has continued to be the primary treatment goal.

GnRHa is the most effective treatment for precocious puberty [21]; it can promote significant regression of sexual characteristics or at least prevents further progression. However, several studies have suggested that monotherapy with GnRHa should no longer be recommended routinely to augment height in adolescents with normal onset of puberty. Furthermore, our study showed that the growth velocity of the GnRHa + rhGH treated group was maintained at a relatively low level during the treatment course. The duration of treatment was much longer than that of the other two groups. Hence, several years of GnRHa + rhGH combinational therapy were necessary to obtain clinically significant increases in height, which was consistent with other studies [22, 23]. Medical insurance in China does not cover treatment with GnRHa/rhGH, and the high cost of therapy could result in a financial burden for the family. This might be why GnRHa combined with rhGH therapy is less common than treatment with AIs combined with rhGH. Thus, the cost of GnRHa combined with rhGH therapy should be weighed against the height gain produced before initiating treatment.

AIs do not inhibit the development of puberty in boys. In addition, the ability to administer AIs orally and their high efficiency makes them realistic alternatives for treating boys with short stature. Our study showed that growth velocity was higher during treatment with AI + rhGH than with the other two treatments. The patient AHts could reach or even exceed the target height (the disparities were 4.13 ± 4.12 cm). However, participants receiving AIs might exhibit symptoms of hyperandrogenism, such as facial acne or oily skin. Although there has not been any published report concerning the influence of AIs on long-term male reproductive ability, the risks and benefits of such treatment need additional prospective long-term outcome analyses.

Our study demonstrated that all three therapy regimens could significantly improve adult height. Interestingly, most parents chose to stop treatment when their children attained a height of 170 cm. We discovered during our follow-up examinations that 170 cm was a generally acceptable height for males in China. If the combinational therapy had been continued, it would have considerable added benefit. However, this was a retrospective clinical study, and additional investigation into the effects of combination therapy on ISS in patients with advanced bone age should be carried out using randomized controlled studies.

### Study limitations

Our retrospective study only reviewed 68 children in China, which was a relatively small number of subjects. Furthermore, the patients were not randomized, and no placebo group was included. Another limitation of this study was that the drug absorption rate and genetic potential 、sex hormones might be associated with the observed differences in height, which could influence the accuracy of model to predict height.

## Conclusions

In conclusion, different therapeutic approaches to improve the adult height of males with ISS and at advanced bone ages were investigated. The optimal strategy remains a matter of debate. Therefore, for children with ISS and at advanced bone age, clinicians should weigh the advantages and disadvantages of the various treatments prior to selecting a treatment protocol.

## Data Availability

All data generated or analyzed during this study are included in this article. Further enquiries can be directed to the corresponding author.

## Abbreviations

rhGH: Recombinant human growth hormone
AIs: Aromatase inhibitors
GnRHa: Gonadotropin-releasing hormone analogue
ISS: Idiopathic short stature
AHt: Adult height
GHD: Growth hormone deficiency
HtSDS-CA: The standard deviation score of the chronological age and height
HtSDS-BA: The standard deviation score of the bone age and height
THt: Target height
PAH: Predicted adult height
AHtSDS: Adult height standard deviation score
PAHSDS: Predicted adult height standard deviation score
